# Magnetic Resonance Imaging of Glucose Uptake and Metabolism in Patients with Head and Neck Cancer

**DOI:** 10.1038/srep30618

**Published:** 2016-07-27

**Authors:** Jihong Wang, Joseph Weygand, Ken-Pin Hwang, Abdallah S. R. Mohamed, Yao Ding, Clifton D. Fuller, Stephen Y. Lai, Steven J. Frank, Jinyuan Zhou

**Affiliations:** 1 Department of Radiation Physics, The University of Texas MD Anderson Cancer Center, Houston, TX, USA; 2 Department of Imaging Physics, The University of Texas MD Anderson Cancer Center, Houston, TX, USA; 3 Department of Radiation Oncology, The University of Texas MD Anderson Cancer Center, Houston, TX, USA; 4 Department of Clinical Oncology and Nuclear Medicine, Faculty of Medicine, Alexandria University, Alexandria, Egypt; 5 Department of Head and Neck Surgery, The University of Texas MD Anderson Cancer Center, Houston, TX, USA; 6 Department of Molecular and Cellular Oncology, The University of Texas MD Anderson Cancer Center, Houston, TX, USA; 7 Division of MR Research, Department of Radiology, Johns Hopkins University, Baltimore, MD, USA

## Abstract

Imaging metabolic dysfunction, a hallmark of solid tumors, usually requires radioactive tracers. Chemical exchange saturation transfer (CEST) imaging can potentially detect and visualize glucose uptake and metabolism, without the need for radioisotopes. Here, we tested the feasibility of using glucose CEST (glucoCEST) to image unlabeled glucose uptake in head and neck cancer by using a clinical 3T magnetic resonance imaging (MRI) scanner. The average CEST contrast between tumors and normal tissue in 17 patients was 7.58% (*P* = 0.006) in the 3–4 ppm offset frequency range and 5.06% (*P* = 0.02) in 1–5 ppm range. In a subgroup of eight patients, glucoCEST signal enhancement was higher in tumors than in normal muscle (4.98% vs. 1.28%, *P* < 0.021). We conclude that glucoCEST images of head and neck cancer can be obtained with a clinical 3T MRI scanner.

Rapidly growing cancer cells typically have much higher rates of glycolysis than do their normal tissues of origin^1^,^2^,^3^,^4^. High rates of glucose uptake in tumors appear as increased activity on ^18^F-fluorodeoxyglucose positron emission tomography (FDG-PET) images, and this is the underlying principle of PET imaging for cancer staging and treatment assessment^5^. Chemical exchange saturation transfer (CEST) imaging^6^,^7^, a noninvasive magnetic resonance imaging (MRI)-based technique, has been used to detect and image glucose uptake and metabolism in mice^8^,^9^,^10^,^11^,^12^,^13^ and shows promise for clinical applications^14^. In animal studies, CEST has been shown to detect glucose concentrations of a few millimoles *in vivo*. Unlike PET/computed tomography (CT), CEST imaging does not involve ionizing radiation, carries little to no risk to the patient, and can be repeated as necessary for cancer diagnosis, staging, and assessment of response.

Although extensive *in vivo* CEST studies have been done with various animal models^15^,^16^,^17^,^18^, studies of CEST using clinical MRI scanners have focused primarily on patients with brain tumors^19^,^20^,^21^ or stroke^22^. CEST imaging in patients with head and neck cancer is particularly difficult because of the complexity of the anatomic structures and the field inhomogeneity artifacts caused by susceptibility and motion^23^. Also, most previous glucose CEST (glucoCEST) imaging studies were performed at ultrahigh field strengths (7T or higher). Here, we adapted an immobilization device from the radiation therapy to image patients with head and neck cancer in the immobilized position (Fig. 1). To the best of our knowledge, this is the first CEST imaging study in which a standard clinical (3T) MRI scanner was used to detect unlabeled glucose uptake in a prospective cohort of patients with human papillomavirus–positive (HPV+) oropharyngeal head and neck cancer.

## Results

### CEST Signals in Head and Neck Cancer Tumors and Surrounding Tissues

**Figure 1:**
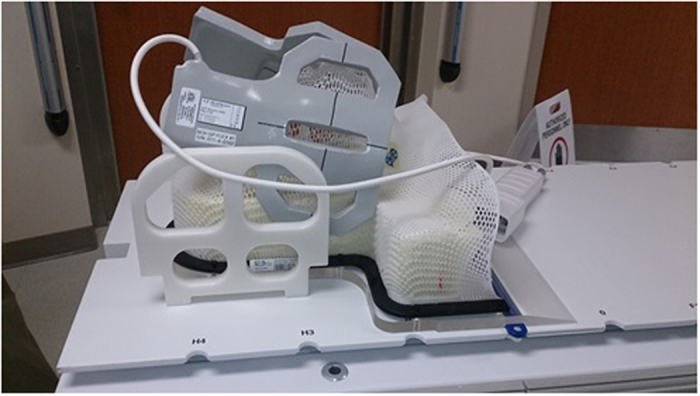
Patient immobilization device and magnetic resonance imaging (MRI) coil arrangement. Imaging patients while immobilized in the position in which they would receive radiation treatment drastically reduces motion artifact, enabling the acquisition of high-quality chemical exchange saturation transfer (CEST) and other MR images.

**Figure 2:**
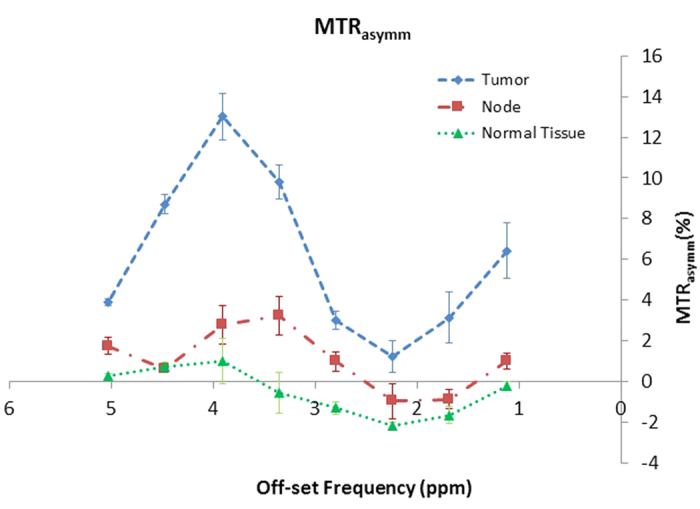
Magnetization transfer ratio asymmetry (MTR_asym_) spectra of tumors, nodes and normal tissues for one patient. The MTR_asym_ of the tumor was higher than that of the normal tissue, particularly in the 3–4 ppm range.

We first performed a CEST study on 17 patients who had been treated for squamous cell carcinoma of the oropharynx. MRI scans were obtained with a Discovery 750 3T MRI scanner system, in which two 6-channel flex coils are placed laterally, centered on the tongue base (Fig. 1), while patients were immobilized in a position in which they would receive radiation treatment^24^. Tumors were located on the T2-weighted fast spin echo images, and a slice through the largest cross-section of the tumor was selected for the CEST imaging measurements. Postprocessing of CEST data was based on asymmetry analysis of the magnetization transfer ratio (MTR) or Z-spectrum of the CEST image series, quantified as MTR_asym_^25^,^26^. A plot of MTR_asym_ against the saturation offset frequency for a patient’s tumor, nodes, and adjacent healthy tissue is shown in Fig. 2. As expected, the MTR_asym_ signal was greater in the tumor regions than in the normal surrounding tissues over the entire saturation frequency offset range.

### Quantitative Analysis of CEST Contrast: Tumors vs. Normal Tissue

**Figure 3:**
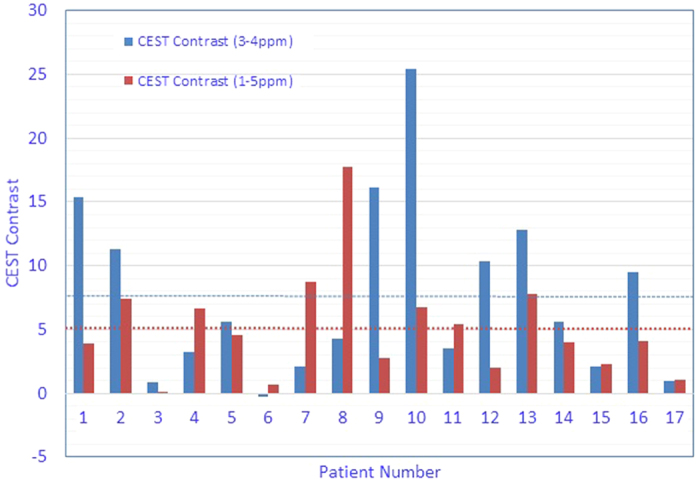
Chemical exchange saturation transfer (CEST) contrast in tumors vs. normal tissues for all patients. The CEST contrast values are the integrated MTR_asym_ signal differences between tumor and normal tissue over the offset frequencies of 3–4 ppm and over 1–5 ppm. The dotted lines indicate the averaged magnitude of CEST contrast over all patients.

For each CEST scan, we integrated the MTR_asym_ spectrum over the offset frequency range of 1 to 5 ppm (the total CEST signal), and we quantified CEST contrast as the area between the MTR_asym_ curves for tumors and healthy regions. The distribution of measured CEST contrasts in tumors for all 17 patients is shown in Fig. 3. The CEST contrast between tumor and normal tissue was evident in most patients, despite variations in the magnitude between patients. In the 1–5 ppm range, the average magnitude of CEST signal in tumor tissue from all 17 patients was found to be higher than that of normal surrounding tissue by 5.06% (*P* = 0.02), a statistically significant difference also noted in a previous report^23^.

**Figure 4:**
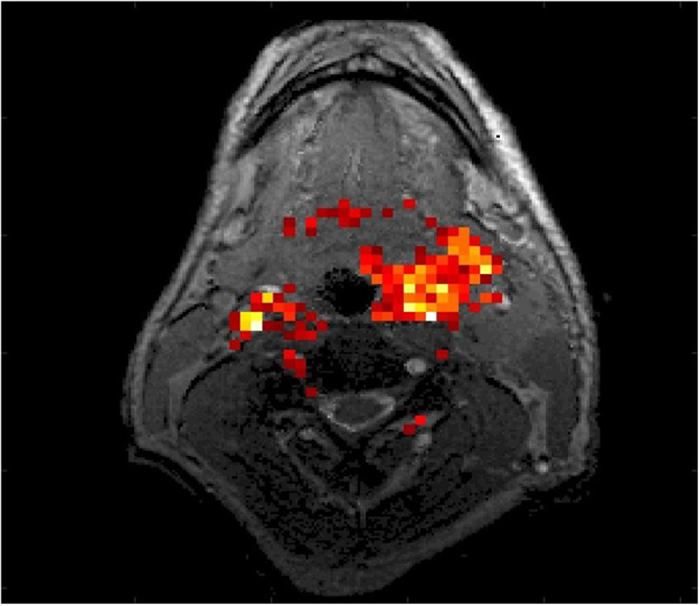
The measured chemical exchange saturation transfer (CEST) contrast value overlaid on an anatomic image. The tumor region showed higher CEST signal (as measured in integrated MTR_asym_ over 1–5 ppm; indicated by red and yellow areas) than the surrounding tissues.

We also measured CEST contrast in the 3–4 ppm range (which may be dominated by the amide proton transfer effect^19^,^20^,^21^). In that range, the average CEST contrast between tumor and normal tissue for all 17 patients was 7.58% (*P* = 0.006), which was larger than the average from the 1–5 ppm range. The integral of MTR_asym_ over the 1–5 ppm frequency range is overlaid on an anatomic image in Fig. 4. The tumor showed higher CEST signal (indicated by red and yellow areas) than the surrounding tissue.

### glucoCEST Enhancement (GCE) in Tumors and Normal Tissue

**Figure 5:**
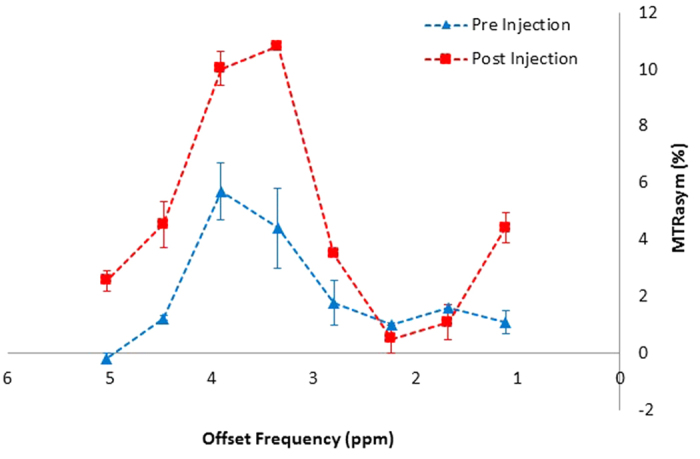
Magnetization transfer ratio asymmetry (MTR_asym_) curves in a tumorous region before and after the injection of unlabeled glucose. Note the higher MTR_asym_ in the 3–4 ppm region and around 1 ppm.

**Figure 6:**
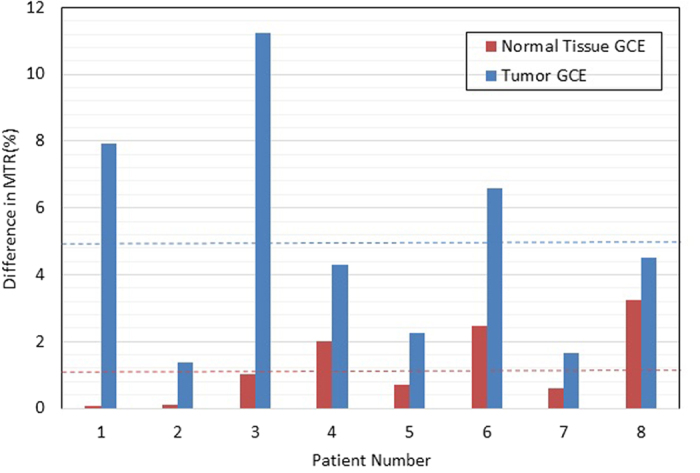
The glucoCEST enhancement (GCE) for tumor and normal tissue regions in all patients. Note the relatively higher GCE in tumor vs. normal tissues. The dotted lines represent the averaged GCE values.

A pair of MTR_asym_ curves in the area of a representative tumor (pre-injection and post-injection) are shown in Fig. 5. Elevated CEST signal in head and neck tumors relative to surrounding normal tissues was clearly seen after the injection of unlabeled glucose, particularly around 1ppm and in the 3–4 ppm range. The fact that glucoCEST appeared in a wide offset range may be due to the relatively fast exchange property of hydroxyl protons with water. The GCE can be calculated by integrating the area between the curves. The GCE values for tumors and normal tissue from all eight patients are shown in Fig. 6. Glucose uptake was reflected in significantly larger increases in GCE in the tumors versus in normal muscle tissues (4.98% in tumor regions vs. 1.28% in muscle, *P* < 0.021). Tumor regions had greater enhancement than the surrounding normal tissue in all patients.

### Comparison of glucoCEST Enhancement and PET Enhancement

**Figure 7:**
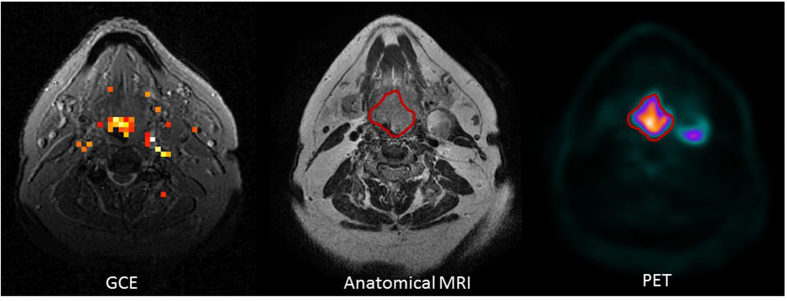
A glucoCEST enhancement (GCE) image with the corresponding anatomic magnetic resonance (MR) image and the positron emission tomography (PET) image. Note that the GCE generally correlated well with the PET results but the spatial distribution of PET activity and GCE did not match exactly.

Finally, we registered patients’ glucoCEST images to their PET/CT images acquired within a week of the CEST MRI scans. For each patient, the PET image slice corresponding to the glucoCEST image was then extracted and qualitatively compared with the GCE image, as shown in Fig. 7. The GCE generally correlated with the PET results, even though the exact spatial distribution of PET activity and GCE did not match well.

## Discussion

Our study showed that the CEST signal (measured in MTR_asym_) in head and neck cancer can be reliably detected and was comparable to that reported for brain tumors^19^,^20^,^21^. Although the exact mechanism underlying the elevation in CEST signal in tumors is complex and not completely understood, one possible explanation is increased cellularity in tumors. Moreover, a reduction of CEST signal (at ~3.5 ppm) has been observed in brain tumors after radiation treatment^16^, which suggests that CEST may be useful for assessing the response of cancer to radiation. Although the underlying mechanism of CEST contrast needs further investigation, the potential translational applications of CEST hold great promise for patients with head and neck cancer, and perhaps for those with other diseases as well.

Our results demonstrate the feasibility and potential utility of glucoCEST for assessing glucose uptake and tumor metabolism in head and neck cancer on a clinical 3T MRI scanner. Indeed, glucoCEST could substantially affect the clinical care of patients with head and neck cancer (and perhaps those with other cancers) because CEST imaging is noninvasive and can be used repeatedly if necessary. If further studies prove that glucoCEST is a reliable alternative to PET, CEST imaging with unlabeled glucose could spare patients the radiation that is by necessity delivered for PET. Our findings further demonstrate that glucoCEST was sensitive enough in patients with head and neck cancer. The glucoCEST MRI scans can be obtained in conjunction with other commonly used MRI sequences within one hour. To address the challenges for clinical glucoCEST imaging from patient motion and susceptibility artifacts, we immobilized the patients in the treatment position by using a mask and dental stent, and in this way we could detect changes in CEST signal on a clinical 3T scanner. Although we did not specifically study biological processes after glucose injection, it is reasonable to speculate that the change in CEST signal or contrast (ranging from 1 ppm to 5 ppm) from baseline to the signal after glucose injection results from the combined effects of glucose uptake and the byproducts of glucose metabolism^8^,^9^,^10^,^11^,^12^,^13^,^14^.

Our results further suggest that glucoCEST could be an alternative to FDG-PET for tumor diagnosis, staging, and treatment assessment. Because CEST is noninvasive and does not use radioisotopes, CEST does not have the limitations of FDG-PET in terms of timing, logistics, and infrastructure. CEST is also more readily available and less expensive than PET. The glucoCEST also differs from FDG-PET in the amount of glucose administered relative to the amount of FDG. In our study, we used 20 mL of glucose, and the sensitivity of detecting glucoCEST was in the millimolar range. FDG-PET uses a much lower concentration of labeled glucose to avoid disturbing the tumor metabolic process. If the glucose concentration used in glucoCEST is large enough to disturb that process; then, it is possible that glucoCEST may not detect actual metabolic processes but instead behaves like a contrast agent. Detailed *in vivo* studies are needed to validate or disprove such concerns. However, many researchers agree that active tumor cells tend to accumulate and demand more glucose as an energy source. Consequently, higher glucose uptake is expected in active tumors than in surrounding normal tissues or in tumors that are less active (possibly as a result of treatment). Therefore, glucoCEST could be used to measure glucose uptake both for staging disease and for assessing its response to treatment.

Like PET images, glucoCEST images have higher contrast in tumor regions, indicating that glucoCEST and PET images likely measure similar metabolic processes in tumors. However, the differences in spatial distribution on the PET and glucoCEST images may indicate that glucoCEST does not measure glucose distribution but instead measures cascade effects and byproducts of the metabolic process after glucose uptake. Future comparative studies are needed to clarify tumor metabolism *in vivo*; nevertheless, the heterogeneities noted in glucoCEST images might be useful for imaging specific metabolites of interest in the future.

Although our study was limited by the relatively small number of patients, our results are still valid and consistent among patients. Our study was also limited in the H&N cancer which as an aggressive tumor, has high glucose metabolism. The exact magnitude of CEST and glucoCEST contrasts should be further quantified in future studies of a larger number of patients and in other disease sites with different glucose metabolism levels and growth rates to generalize the use of this technique. Further research also is needed to understand the contrast mechanism of glucoCEST. The dynamic characteristics of unlabeled glucose *in vivo* should be studied to determine the optimal amount of glucose to inject and the optimal timing of CEST imaging. A detailed dynamic study of glucose uptake at various concentrations using CEST may reveal more information about the mechanism of GCE and possibly the underlying biology of glucose uptake in head and neck tumors. Future studies also should explore CEST contrast variations in patients before, during, and after treatment to determine CEST’s ability to assess treatment response. A study comparing GCE contrast with PET imaging before, during, and after treatment could also reveal some mechanistic differences between the uptake of FDG and unlabeled glucose and perhaps could shed light on *in vivo* tumor metabolic processes.

The peak uptake time for involved nodes may be different from that of tumor; thus, the dynamic study of glucoCEST may have to be done with careful consideration of motion during a long MRI study between the pre- and post-glucose injection. In such a case, image coregistration may be necessary. In the case of tumor sites which have a lot of inherent motion (e.g. lung and liver), motion gating in the imaging acquisition (e.g. respiratory gating) in CEST and glucoCEST must be implemented to reduce motion-related artifact. In the case of small tumor and nodes, partial volume effect might reduce the glucoCEST sensitivity in which case thinner imaging slices may have to be used.

In conclusion, our results show that glucoCEST imaging of patients with head and neck cancer is feasible with a 3T clinical scanner. The fact that tumors have significantly higher CEST signal intensities and higher uptake of unlabeled glucose than does normal tissue makes CEST a potential alternative to PET.

## Methods

### Patients

All experiments were approved by the University of Texas MD Anderson Cancer Center institutional review board (IRB) and were performed in accordance with the IRB rules and policies. Between October 2013 and July 2015, 17 patients with head and neck squamous cell carcinoma of the oropharynx were recruited in this study. All patients gave study-specific informed consent to participate in the study and all experiments were carried out in accordance with the relevant guidelines. Other inclusion criteria were (i) histologically documented stage III or IV HPV-positive squamous cell carcinoma of the oropharynx and (ii) at least one lesion (primary tumor or metastatic node) measuring more than 1.5 cm in its maximum axial diameter.

### MRI Experiments

All MRI studies were done with a Discovery 750 3T MRI scanner system (GE Healthcare, Milwaukee, WI), using two laterally placed 6-channel flex coils centered on the tongue base (Fig. 1). Patients were immobilized in the position in which they would receive radiation treatment with an intraoral tongue-immobilizing/swallow-suppressing dental stent and with a customized thermoplastic head and shoulder mask (Klarity Medical Product, Newark, OH). Patient setup and scanning technique are described in detail elsewhere^24^. This immobilization approach greatly improves image co-registration in longitudinal scans and reduces interval physiologic motion (e.g., swallowing). Scan volume (field of view, 25.6 cm; slice number, ~30; slab thickness, 12 cm; and pixel size, 1 mm × 1 mm in-plane) was prescribed for a standardized spatial region encompassing the palatine process region and the cricoid cartilage for all pre-, mid-, and post-treatment scans. T2-weighted, pre-contrast T1-weighted, and post-contrast T1-weighted with fat saturation axial images were acquired using a fast spin echo sequence.

### CEST MRI

The CEST imaging pulse sequence consisted of a magnetization transfer prepared spoiled gradient echo sequence modified to saturate a range of frequency offsets, producing a spectrum for each voxel that can be used to generate CEST contrast. An off-resonance saturation pulse with a near-rectangular Fermi pulse profile and a duration of 48 ms was applied for each repetition of the sequence. The saturation pulse was applied with frequency offsets in steps of 71.4 Hz over a range of −1000 Hz to 1000 Hz for a total of 29 offsets. We acquired a single-slice CEST image (field of view, 240; slice thickness, 5 mm; repetition time/echo time, 64/4.4; flip angle, 20; number of excitations, 1 cm; acquisition matrix, 192 mm × 192 mm; and bandwidth, 244.14 Hz/pixel) for each patient, and the scan time was approximately 5 minutes for a Z-spectrum of 29 saturation frequencies covering −1000 to 1000 Hz relative to the bulk water resonance frequency (defined as ∆f = 0). Tumors were located on the T2-weighted fast spin echo images, and a single slice through the largest cross-section of the tumor was selected for the CEST imaging measurements.

### CEST Data Analysis

Post-processing software was developed with IDL (Exelis, Boulder, CO) and was based on asymmetry analysis of the Z-spectrum as reported elsewhere^24^,^25^. The MTR curve (or Z-spectrum) was obtained from the CEST image series. In particular, the asymmetric MTR (MTR_asym_) at a given offset ∆f was defined as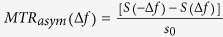. This was calculated in regions of interest encircling the tumor, nodes, and adjacent healthy tissue over the entire offset frequency range.

For each CEST scan, we obtained the total CEST signal by integrating the MTR_asym_ spectrum over the offset frequency range of 1 to 5 ppm. We further quantified the CEST contrast as the area between the MTR_asym_ curves for tumors and healthy regions. This value was then normalized by the length of the integration region to convert the contrast to a percentage difference:


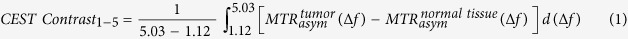


Because previous *in vivo* studies indicated that amide protons resonate at a frequency of 3.5 ppm^25^,^26^, we also measured the CEST contrast in the 3–4 ppm range (which may be dominated by the amide proton transfer effect^19^,^20^,^21^). The CEST contrast between the tumor and the normal region in this ppm range was calculated by integrating the MRT curves as follows:


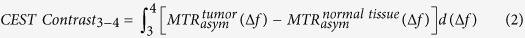


### Glucose CEST MRI

During the glucose enhancement experiment, a baseline CEST image was obtained from 8 patients after the T1-weighted and T2-weighted images. Then, a bolus of unlabeled glucose (20 mL) was injected intravenously, and a second CEST scan was obtained 10 minutes later to study glucose CEST (glucoCEST) enhancement. This timing encompasses the peak glucose uptake^8^,^9^,^10^,^11^,^12^,^13^,^14^. Our CEST sequence took 5 minutes to complete; therefore, this acquisition timing should ensure the capture of peak glucose uptake in the tumor.

Asymmetric MTR was analyzed in both sets of the acquired CEST image series (baseline and after glucose injection). As was done previously in an animal study^8^, glucoCEST enhancement (GCE) was defined as the area between the two MTR_asym_ curves, before and after glucose injection. Again, we chose to integrate the MTR_asym_ curve over the 1–5 ppm range because these offset frequencies are all relevant to the metabolic processes in a tumor^8^,^9^,^10^,^11^,^12^,^13^,^14^. This value was then normalized by the length of the integration region to convert the results to percentages:


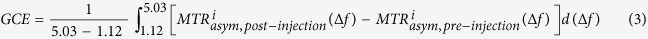


Here, i denotes that this parameter can be calculated for either tumor or healthy regions.

### Comparison of GCE and PET

Patients’ GCE images were first registered to their anatomic (T1/T2) images acquired in the same MRI study. Then, the anatomic MRI study (T1/T2) was registered to the patient’s PET/CT images, which had been acquired within 1 week of the MRI/CEST, and the PET image slice corresponding to the GCE image was extracted. Finally, the GCE color map image was overlaid on the anatomic MRI image and qualitatively compared with the corresponding PET image.

## Additional Information

**How to cite this article**: Wang, J. *et al*. Magnetic Resonance Imaging of Glucose Uptake and Metabolism in Patients with Head and Neck Cancer. *Sci. Rep*. **6**, 30618; doi: 10.1038/srep30618 (2016).
